# Plasma Biochemistry, Intestinal Health, and Transcriptome Analysis Reveal Why Laying Hens Produce Translucent Eggs

**DOI:** 10.3390/ani14172593

**Published:** 2024-09-06

**Authors:** Yuanjun Du, Dan Hao, Wei Liu, Wei Liu, Dapeng Li, Qiuxia Lei, Yan Zhou, Jie Liu, Dingguo Cao, Jie Wang, Yan Sun, Fu Chen, Haixia Han, Fuwei Li

**Affiliations:** 1 Jinan Key Laboratory of Poultry Germplasm Resources Innovation and Healthy Breeding, Shandong Academy of Agricultural Sciences, Poultry Institute, Jinan 250100, China; 2 Shandong Technology Innovation Center of Laying Hens, Jinan 250102, China; 3 College of Veterinary Medicine, Qingdao Agricultural University, Qingdao 266109, China; 4 Shandong Blue Horizon Ecological Agriculture Co., Ltd., Jinan 250100, China

**Keywords:** eggshell translucency, laying hen, intestinal function, transcriptome

## Abstract

**Simple Summary:**

The precise structure and function of the intestinal tract, along with the underlying molecular mechanisms governing the production of translucent eggs, remain unclear. The present study revealed that translucent eggs exhibited a thicker eggshell and a lower egg yolk color, which may be attributed to a disruption in the plasma lipid metabolism of the laying hens, a reduction in their intestinal antioxidant capacity, a decline in digestive and absorptive processes, and an alteration in their metabolic capabilities. Furthermore, we identified a total of 471 differentially expressed genes (DEGs) in duodenal tissue from laying hens producing translucent and normal eggs, comprising 327 upwardly expressed genes and 144 downwardly expressed genes. Enrichment analysis revealed that the up-regulated genes were predominantly enriched in metabolism-associated pathways and oxidative phosphorylation pathways, offering novel insights for enhancing egg quality in commercial poultry production.

**Abstract:**

Producing translucent eggs has been found to reduce the quality and safety of the eggs, as well as the demand from consumers. However, the intestinal function and the molecular mechanism for the production of translucent eggs remain uncertain. A total of 120 eggs from 276-day-old Jining Bairi were divided into two groups based on eggshell translucence: the translucent egg group (group T) and the normal group (group C). Group T exhibited thicker eggshells and a lower egg yolk color. Subsequently, we divided the chickens into translucent and normal groups based on their egg quality. We then assessed the plasma biochemical index, intestinal morphology and structure, enzyme activity, and antioxidant capacity of the hens producing translucent eggs compared to those producing normal eggs. The results showed that the ratio of duodenal villus length to crypt depth, succinate dehydrogenase (SDH) activity, chymotrypsin, total ATPase (T-ATPase), alkaline phosphatase (AKP), and glutathione peroxidase (GSH-Px) activities were decreased in the hens produced translucent eggs (*p* < 0.05), but malondialdehyde (MDA) content was increased (*p* < 0.05); jejunal lipase activity, Na^+^K^+^-ATPase activity, total antioxidant capacity (T-AOC), and GSH-Px activities were decreased (*p* < 0.05) in group T; ileal amylase and Ca^2+^Mg^2+^-ATPase activities were also decreased (*p* < 0.05) in group T. In addition, we identified a total of 471 differentially expressed genes (DEGs) in duodenal tissue, with 327 up-regulated genes and 144 down-regulated genes (|log_2_FC| ≥ 1 and *p* < 0.05). Enrichment analysis showed that the up-regulated genes, such as *GSTT1*, *GSTO2*, and *GSTA3*, were mostly enriched in metabolism of xenobiotics by cytochrome P450, drug metabolism-cytochrome P450, and oxidative phosphorylation pathways. The results of our study indicate that plasma lipid metabolism disorder, decreased intestinal antioxidant capacity, and altered intestinal metabolism capabilities may influence the formation of translucent eggs.

## 1. Introduction

Eggshell translucency refers to the appearance of transparent spots on the shell due to moisture accumulation, especially when light penetrates the egg [1]. This phenomenon varies considerably between breeds and even between individual eggs [2,3]. Translucent eggs not only increase the risk of bacterial contamination [4], but also reduce consumer demand, ultimately leading to a decrease in economic profitability for producers [5].

The intestinal tract is a crucial component of the avian digestive apparatus, responsible for nutrient digestion and absorption [6,7], lipid metabolism, detoxification, neuroendocrine function, and immune and defensive functions against external pathogens [8,9]. Numerous studies have demonstrated significant correlations among the capacity of the intestine to absorb nutrients, particularly calcium ion, and egg production, egg quality, and eggshell quality [10,11,12,13,14]. One crucial reason for the hens laying translucent eggs may be attributed to a corresponding impairment of their intestinal functional state [15]. To date, comprehensive molecular mechanism differences in intestinal functions of laying hens producing distinctively different quality eggs remain poorly understood.

The intestinal segments exhibit a range of absorptive capacities for various nutrients and minerals, including calcium [16]. Furthermore, calcium constitutes approximately half of the elemental composition of eggshells. Consequently, the quality of eggshells is directly correlated with the utilization of calcium by laying hens [17]. In the intestine, calcium ions are absorbed mainly through active transcellular processes and passive paracellular diffusion [18,19,20,21]. After absorption, calcium is transported into the bloodstream, contributing to blood calcium levels. Subsequently, the calcium travels to the uterine portion of the oviduct, where the eggshell is formed. A series of chemical reactions results in the formation of calcium ions into calcium carbonate, which is subsequently deposited on the eggshell membrane to form the eggshell.

It was postulated that alterations in intestinal morphology, structure, and function could influence the absorption and transport of calcium ions, leading to reduced calcium ion deposition and the formation of translucent eggs. To verify this, we conducted egg quality tests, assessed the plasma biochemical indexes, intestinal morphology and structure, intestinal enzyme activity, and intestinal antioxidant capacity, and analyzed the duodenum transcriptome of laying hens who produced translucent and normal eggs. Our study will provide a framework for future research into the production of less translucent eggs.

## 2. Materials and Methods

### 2.1. Ethics Statement

The study was carried out in compliance with the Animal Research: Reporting of in vivo Experiments (ARRIVE) guidelines. The care and use of experimental animals were carried out in accordance with the Directory Proposals on the Ethical Treatment of Experimental Animals, established by the Ministry of Science and Technology (Beijing, China). All study animal procedures were approved and supervised by the Animal Ethics committee of the Shandong Academy of Agricultural Science (Permit No: 2018412). All methods were performed in accordance with relevant guidelines and regulations.

### 2.2. Animal and Sample Collection

**Figure 1:**
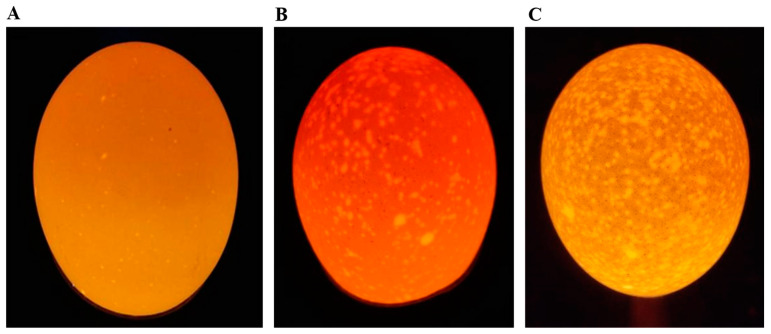
Translucent egg grading. (**A**) First-class translucent egg. (**B**) Second-class translucent egg. (**C**) Third-class translucent egg.

One hundred 276-day-old Jining Bairi hens were randomly selected from the Poultry Institute of the Shandong Academy of Agricultural Sciences, Shandong, China. Chickens were provided with feed and watered *ad libitum*, under the same management conditions, with the same feeding environment and same composition of feed. The laying hens were raised in single cages after numbering. We collected eggs from each hen daily at 09:00 over a 35-day period and stored them in a controlled environment (temperature 20 to 25 °C, relative humidity 50 to 60%) for 1 day. We classified the eggs into 3 grades according to the degree of shell translucence: first-class: opaque; second-class: semi-translucent; and third-class: translucent [1,22] (see Figure 1). All eggs were examined using a conventional illumination source (Feisheng Electronic Technology Co., LTD., Shanghai, China) [1,22]. Subsequently, we selected the top ten hens that produced the highest rate of normal eggs to serve as the control group (group C), and ten laying hens that produced the highest rate of translucent eggs were selected as the translucence group (group T).

The middle section of the duodenum, jejunum, and ileum tissues from laying hens in both groups were fixed in 4% neutral formaldehyde solution, and then frozen in liquid nitrogen and stored in −80 °C. Measurements of egg length (L) and width (W) were taken with a digital caliper to the nearest 0.01 mm. The egg shape index (SI) was determined from the measurements according to the formula from Anderson et al. [23].

$$\mathrm{SI}=\left(\frac{W}{L}\right)\times 100$$

We weighted the yolk weight (YW) after separating it from the albumin, and compare it with the egg weight (EW) to obtain the egg yolk ratio (EYR). EYR was calculated using the following formula:

$$\mathrm{EYR}=\left(\frac{YW}{EW}\right)\times 100$$

We removed the eggshell membrane and dried the eggshells; we then used an electronic tray balance to obtain the egg shell weight (ESW), and calculated the egg shell ratio based on the following formula:

$$\mathrm{ESR}=\left(\frac{ESW}{EW}\right)\times 100$$

The blunt, middle, and sharp shell thickness were tested using the Egg Shell Thickness Gauge (Fujihira Industry Co., Ltd., Tokyo, Japan) with the membrane. Eggshell thickness was calculated by the average of blunt, middle, and sharp shell thickness. Eggshell strength was measured using an Egg Force Reader (Tenovo International Co., Limited, Beijing, China) and egg weight was measured using an Egg Quality Analyzer (Robotmation Co., Ltd., Tokyo, Japan). We also cracked the eggs and poured their contents into a designated position with the yolk centered to assess the eggs’ quality using an Egg Quality Analyzer (Robotmation Co., Ltd., Tokyo, Japan). This evaluation included measurements of albumen height, yolk color, and the Haugh unit. The eggshell calcium, phosphorus, and ash content were determined by Shandong Runda Testing Technology Co., Ltd. (Jinan, China).

### 2.3. Intestinal Tissue Morphology

The duodenum, jejunum, and ileum tissues were fixed with 4% paraformaldehyde for 72 h. After fixation, paraffin embedding and hematoxylin and eosin (HE) staining were performed by Wuhan Servicebio Technology Co., Ltd. (Wuhan, China). Well-formed villi with a clear field of view on each slice were selected for scanning using an inverted microscope (Mack Photoelectric Instrument Co., Chongqing, China).

Villus height was measured from the tip to the base of the villus, and crypt depth was measured from the base of the villus to the mucosa. Measurements of villus height and crypt depth were taken only from sections where the plane of section ran vertically from the tip of villus to the base of an adjacent crypt, using a microscope (Mack Photoelectric Instrument Co., Chongqing, China).

Ten well-oriented villus sections from three different locations on each hen were used to determine the indices. After measured each villus height and crypt depth, the ratio of villus height to crypt depth was calculated.

### 2.4. Plasma Biochemical Index

We tested the plasma biochemical index including total antioxidant capacity (T-AOC) (catalog numbers: A015-3-1), catalase (CAT) (A007-1-1), glutathione (GSH-Px) (A005-1-2), superoxide dismutase (SOD) (A001-3-2), triglyceride (TG) (A110-2-1), malondialdehyde (MDA) (A003-1-2), low-density lipoprotein cholesterol (LDL-C) (A113-1-1), high-density lipoprotein cholesterol (HDL-C) (A112-1-1), phosphorus (C006-1-1), and calcium (C004-2-1). All indexes were tested using the commercially available kit purchased from Nanjing Jiancheng Bioengineering Institute (Nanjing, China).

### 2.5. Intestinal Biochemical Indexes

We assessed the activity of three intestinal digestive enzymes, four intestinal energy metabolism enzymes, and five antioxidant factors in the duodenum, jejunum, and ileum. The digestive enzymes tested were amylase (C016-1-1), lipase (A054-1-1), and chymotryps (A080-3-1) in each intestinal segment. Energy metabolism enzymes, namely, natrium potassium ATPase (Na^+^K^+^-ATPase) activity (A070-6-2), calcium and magnesium ATPase (Ca^2+^Mg^2+^-ATPase) activity (A070-6-2), ATPase (A095-1-1), alkaline phosphatase (AKP) activity (A059-2-2), and succinate dehydrogenase (SDH) activity (A022-1-1), were also measured following the manufacturer’s instructions. The five antioxidant capacity indexes, namely, T-AOC, MDA, CAT, GSH-Px, and SOD, were also tested. All commercially available kits were purchased from Nanjing Jiancheng Bioengineering Institute (Nanjing, China).

After removing intestinal contents, each intestinal tissue segment was homogenized (1:4, weight [g]/volume [mL]) with ice-cold sodium chloride solution (154 mmol/L) using a mechanical homogenizer (Shanghai, China) to produce a 20% tissue homogenate. The homogenate was then centrifuge at 2500 rpm for 10 min, and the supernatant was used for the measurements.

### 2.6. Total RNA Isolation and cDNA Library Construction and Sequencing

RNA was extracted and purified from duodenum using TRizol (Invitrogen, Carlsbad, CA, USA) according to the manufacturer’s protocols. RNA quality and purity were assessed using a NanoDrop ND-1000 (ThermoFisher, Waltham, MA, USA). RNA integrity was evaluated using the Bioanalyzer 2100 (Agilent, Santa Clara, CA, USA).

Specific mRNA was captured by PolyA using oligo dT (Invitrogen, Carlsbad, CA, USA), and then fragmented at high temperature with the NEBNext^®^ Magnesium RNA Fragmentation Module (NEB, Ipswich, MA, USA). First-strand cDNA was synthesized from fragmented RNA using the Invitrogen SuperScript™ II Reverse Transcriptase kit (Invitrogen, CA, USA). RNA was removed from the cDNA-RNA hybrid using *E. coli* DNA polymerase I (NEB, Ipswich, MA, USA) and RNase H (NEB, Ipswich, MA, USA) to generate double-stranded cDNA.

To create blunt ends on double-stranded cDNA, dUTP solution (NEB, Ipswich, MA, USA) was added. A base was ligated to the T base adaptor at both ends of the cDNA, with fragments screened and purified using oligo dT. Uracil-DNA glycocasylase (NEB, Ipswich, MA, USA) was used to digest the second-strand cDNA, resulting in a library of 300 base pair (bp) ± 50 bp fragments which was then amplified by PCR. Finally, paired-end sequencing was performed using Illumina Novaseq™ 6000 (Illumina, San Diego, CA, USA) according to standard operating procedures using the PE150 sequencing.

### 2.7. Real-Time Fluorescence Quantitative PCR

Seven differentially expressed genes (DEGs), namely, microsomal glutathione S-transferase 1 gene (*MGST1*), *MGST2*, ATP binding cassette subfamily G member 8 (*ABCG8*), *ABCG5*, fatty acid 2-hydroxylase (*FA2H*), NAD(P)H quinone dehydrogenase 1 gene (*NQO1*), and coagulation factor II thrombin receptor (*F2R*), were randomly selected to confirm the accuracy of sequencing results using qRT-PCR, with *β-Actin* as the reference gene.

Primers were designed using Primer Express 3.0 software according to the mRNA sequence from GenBank, and were synthesized by Sangon Biotech (Shanghai, China). The primers are listed in Table S1. cDNA was synthesized using the Evo M-MLV reverse transcription premix kit (Accurate Biology, Changsha, China). Quantitative PCR was performed using the SYBR Green *Pro Taq* HS premix qPCR kit (Accurate Biology, Changsha, China) on a real-time fluorescent quantitative PCR apparatus (Roche, Basel, Switzerland).

The PCR condition was as follows: initial denaturation 95 °C for 30 s, followed by 40 cycles of 95 °C for 5 s and 60 °C for 34 s. Relative gene expression levels were calculated using the 2^−△△Ct^ method [24], which normalized to *β-Actin* expression.

### 2.8. Bioinformatics Analysis

Raw data were stored with FASTA format. To generate high-quality data, we used CUTADAPT software (version 1.9) [25]. Clean reads were aligned with the chicken genome reference using Hisat2 (version 2.0.4) [26]. Transcript reconstruction and gene expression levels in samples were determined using StringTie software (version 1.3.4d) [26]. Gene expression was quantified as fragments per kilobase of transcript sequence per millions base pairs sequenced (FPKM)

$$\mathrm{FPKM}=\frac{Total\;exon\;fragments}{mapped\;reads\left(millions\right)\times exon\;length\;(kb)}$$

Differential expression analysis was performed using DESeq2 (version 1.42.1) [27]. Gene Ontology (GO) terms and Kyoto Encyclopedia of Genes and Genomes (KEGG) enrichment analyses were conducted with the R package *clusterProfiler* (version 4.0) [28], based on the Benjamini–Hochberg method with statistical significance set at an adjusted *p*-value less than 0.05. Functional enrichment was visualized using the R package *enrichplot* (version1.15.2) [29].

### 2.9. Data Statistics and Analysis

Statistical analysis was performed using SPSS 20.0 software, with an independent samples *t*-test employed to compare the two groups. The results are expressed as mean ± standard error. A *p*-value < 0.05 indicates a significant difference, while a *p*-value < 0.01 shows a very significant difference.

## 3. Results

### 3.1. Egg Quality Parameters and Hen Plasma Biochemical Index

Table 1 presents a comprehensive overview of the egg quality parameters and the plasma biochemical indices of the laying hens. The eggshell thickness and ratio significantly increased (*p* < 0.01) in group T, while the egg yolk color significantly decreased (*p* < 0.05) in group T (Table 1). The egg weight was slightly increased, whereas the eggshell index, eggshell color, Haugh unit, and albumen height tended to decrease in group T (Table S2). The eggshell strength and EYR were no different between the two groups (Table S2). The calcium content was slightly increased in group T; however, the phosphorus and ash content in the eggshell was slightly decreased in translucent eggs. The plasma biochemical indexes, including LDL-C, HDL-C, phosphorus, and calcium, exhibited a significant decrease, whereas MDA demonstrated an increase (*p* < 0.05) in group T (Table 1). T-AOC, CAT, GSH-PX, and SOD were found to be slightly reduced in the hens that laid translucent eggs, while the TG value exhibited a tendency towards elevation (Table S2).

### 3.2. Intestinal Morphology and Structure, Enzyme Activity, and Antioxidant Capacity

To obtain a comprehensive understanding of the digestive and absorptive capabilities within the small intestine, we analyzed the intestinal morphology, structure, digestive enzyme, and energy metabolism enzyme activity, as well as the antioxidant capacity (Table 2). Our findings demonstrated a decline in intestinal villus length and an upward trajectory in crypt depth (Table S2), which resulted in the ratio of villus length to crypt depth in the duodenum exhibiting a decline in group T (*p* < 0.05). Subsequently, the intestinal digestive enzyme activity and energy metabolism enzyme activity results showed that chymotrypsin activity diminished (*p* < 0.05) in the duodenum of group T, and lipase activity in the jejunum extremely decreased (*p* < 0.01). Moreover, amylase activity in the ileum decreased (*p* < 0.05) in group T. In the duodenum tissue of group T, SDH activity decreased (*p* < 0.01), as did total ATPase and AKP activity (*p* < 0.05). In contrast, only Na^+^K^+^-ATPase activity was decreased in the jejunum. Similarly, Ca^2+^Mg^2+^-ATPase activity exhibited a reduction (*p* < 0.05) within the same group in the ileum. Significant reductions in intestinal antioxidant capacity, such as GSH-Px activity in the duodenum and jejunum (*p* < 0.05), were also found. In contrast, MDA content exhibited an increase in the duodenum (*p* < 0.05).

### 3.3. Quality Control and Statistic Alignment for RNA Sequencing Data

In order to reveal the molecular mechanism of intestinal effects on the formation of translucent egg, we conducted transcriptome analysis to detect the differentially expressed genes in duodenum from the two groups. The results of the transcriptome sequencing of the duodenal tissues from the hens that laid translucent eggs and normal eggs are shown in Table S4. The total sequencing data for each sample were 40,786,044 to 55,668,976. The average GC content was 51.13%, and the percentage of the Q30 base was more than 97.00%. After filtering, the number of clean reads per sample ranged from 38,508,396 to 52,293,940. The total mapped reads were above 82%, and the unique mapped reads were between 61.62% and 69.28% when compared with the chicken reference genome (Table S5). The higher mapped rate indicated that the RNA-Seq data quality meets the requirements for analyzing.

### 3.4. Screening and Validation of Differentially Expressed Genes between Groups

**Figure 2:**
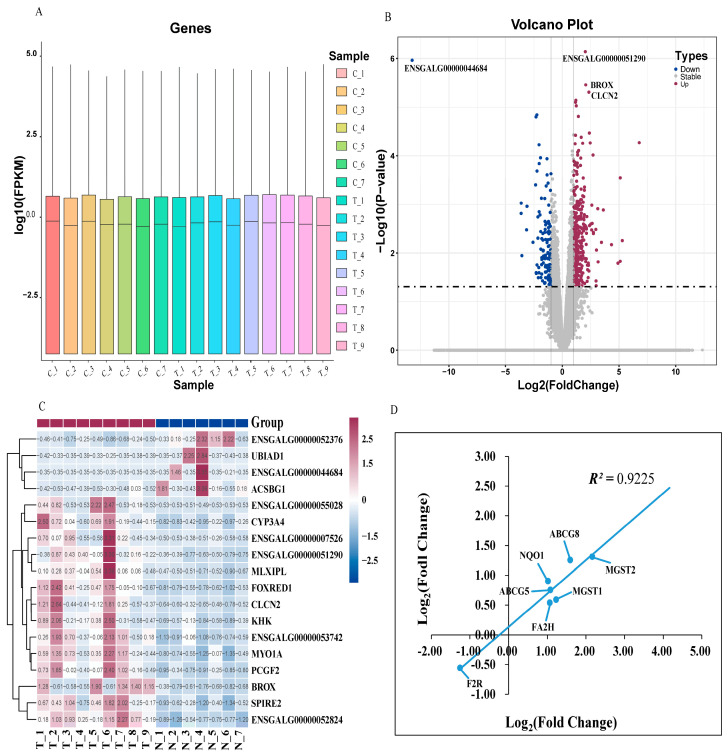
Transcriptome analysis. (**A**) The gene density distribution of each sample according to Log_10_ (FPKM). (**B**) The volcano plot depicts differentially expressed genes between group C and group T. The DEGs are shown as blue (down) and red (up) dots based on *p* < 0.05 and Log_2_ (Fold Change) ≥ |1|, while the gray dots represent genes with no significance. (**C**) Hierarchical clustering analysis of the significantly DEGs based on the z-score of the FPKM value. The red color indicates a highly expressed gene and blue indicates a low-expressed gene. (**D**) qRT-PCR validation of RNA-seq results. The Pearson correlation coefficient is labeled as *R*^2^. Log_2_(Fold Change) in the x-axis equals ^−ΔΔCt^ for each comparison. The average cycle threshold (Ct) value for each group is the mean of samples in the group. The average expression of *β-Actin* was used for the normalization of Ct values.

The gene density distribution according to FPKM did not show a difference among the samples (Figure 2A). A total of 23,910 genes were identified, and 471 DEGs were obtained (*p* ≤ 0.05 and |Log_2_ Fold Change| ≥ 1). Among the DEGs, 327 genes were up-regulated and 144 genes were down-regulated (Figure 2B). Furthermore, the hierarchical clustering analysis indicated that the expression trends in the replicates in DEGs (*p* ≤ 0.05 and |log_2_ fold change| ≥ 1) were basically consistent, further proving that our sequencing data had good biological repeatability (Figure 2C). The correlation coefficient between the RNA-Seq and the qRT-PCR of the seven DEGs was 0.9225 (Figure 2D), indicating that the sequencing results were reliable.

### 3.5. Functional Enrichment Analysis of Differentially Expressed Genes

**Figure 3:**
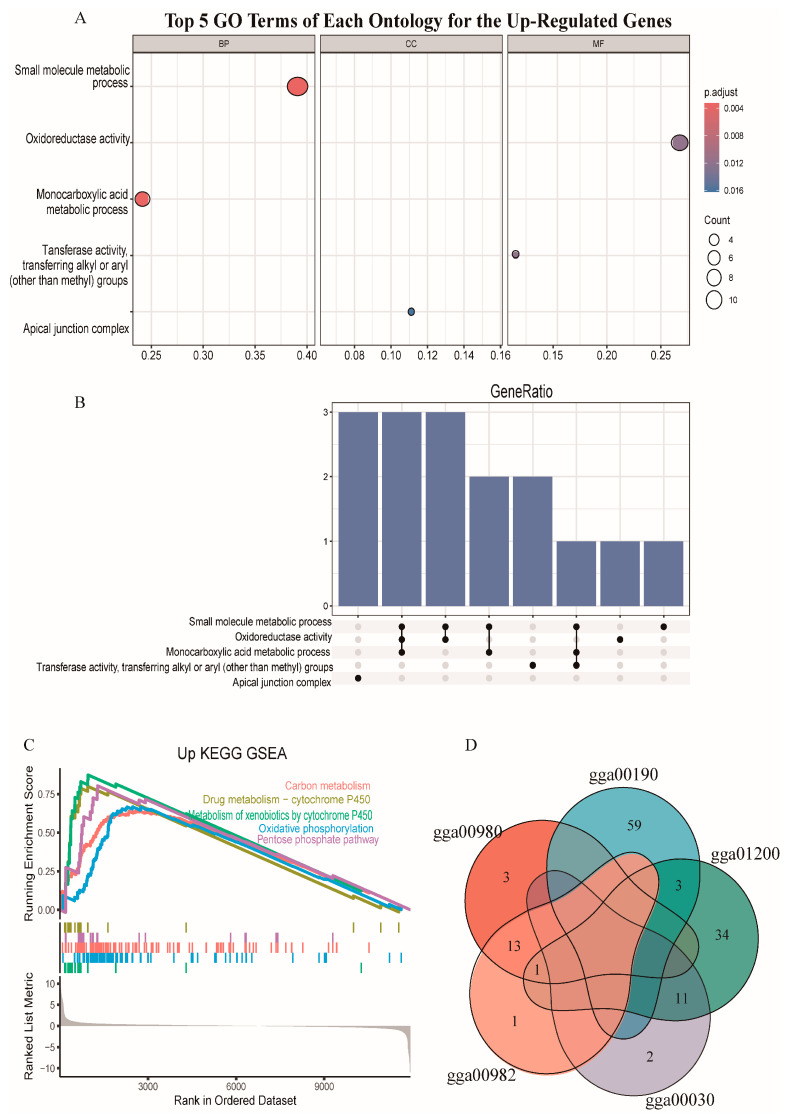
Functional enrichment. (**A**) GO terms of each ontology for the up-regulated genes. The dot size indicates the number of differential genes. The y-axis and x-axis indicate functional pathways and rich factor. (**B**) UpSet plot of interactions for the genes enriched in the top five GO terms. (**C**) Gene Set Enrichment Analysis (GSEA) of the top five KEGG pathways for the up-regulated genes. KEGG: Kyoto Encyclopedia of Genes and Genomes. (**D**) Venn plot for the genes enriched in different pathways.

We conducted GO and KEGG pathway analysis. The GO analysis showed that up-regulated genes were mainly enriched in small molecule metabolic process (GO:0044281), oxidoreductase activity (GO:0016491), and monocarboxylic acid metabolic process (GO:0032787), but the down-regulated genes were not significantly enriched in any GO terms (Figure 3A). Three up-regulated genes, namely, acyl-CoA dehydrogenase family member 11 gene (*ACAD11*), alcohol dehydrogenase 1C (class I) gamma polypeptide gene (*ADH1C*), and aldehyde dehydrogenase 1 family member A1 gene (*ALDH1A1*), were enriched in three different GO terms (Figure 3B). The KEGG enrichment analysis showed that up-regulated genes were mainly enriched in the carbon metabolism (gga01200), drug metabolism-cytochrome P450 (gga00982), metabolism of xenobiotics by cytochrome P450 (gga00980), oxidative phosphorylation (gga00190), and pentose phosphate pathway (gga00030) (Figure 3C), but the down-regulated genes were not significantly enriched in any KEGG pathways. Eleven up-regulated genes were enriched in the pentose phosphate pathway (gga00030) and carbon metabolism (gga01200). Fourteen up-regulated genes, namely, *ADH4*, *ADH6*, *ADH1C*, *GSTA3*, *GSTA4*, *GSTK1*, *GSTM2*, *GSTO2*, *GSTT1*, *HPGDS*, *MGST1*, *MGST2*, *MGST3*, and *UGT1A1*, were enriched in both gga00982 and in gga00980 (Figure 3D).

## 4. Discussion

The formation of translucent eggs is a complex process. Eggshell translucency is regulated by genetic factors with a heritability of 0.18 to 0.22 [2]. Other factors, such as the temperature and humidity of the environment [30], the diet [31], and the age of laying hens [32], could also lead to eggshell translucency. In this study, we divided the chickens into two groups based on the quality of their eggs, and then tested the plasma biochemical indexes, the intestinal morphology and structure, and the intestinal enzyme activity and antioxidant capacity of the two groups of laying hens. A transcriptome analysis of the duodenum from the laying hens who produced translucent and normal eggs was used to explore the DE genes and functional enrichment analysis.

### 4.1. Translucent Eggs Showed Thicker Eggshell and Lower Egg Yolk Color

Eggshell thickness is one of the most important indices of the external characteristics of eggs. The damage rate of eggshells is directly related to small changes in eggshell thickness. Previous researchers have confirmed that translucent eggs have higher eggshell thickness [22,33], which is consistent with our findings. Researchers also have found that translucent egg grade was positively associated with eggshell structure including mastoid space height, mastoid space width, and mastoid space area [30]. Thus, we speculated that a larger mastoid space correlates with a thicker eggshell and a higher translucent score. Additionally, the eggshell ratio significantly increased in the translucent group, likely due to the increased eggshell thickness contributing to a higher eggshell weight. Previous researchers also found that there was no significant difference in egg weight between the two kinds of eggs [22], which is consistent with our findings. Moreover, the lighter egg yolk color in the translucent eggs may be related to differences in the intestinal absorption and transformation ability of carotenoids [34,35,36].

### 4.2. Plasma Lipid Metabolism Disorder Results in Hens Laying Translucent Eggs

TC, TG, HDL-C, and LDL-C in serum are closely related to lipid metabolisms [37]. HDL-C and LDL-C content in the plasma of the T group was significantly reduced, while MDA content was significantly increased. This indicates that plasma lipid metabolism in laying hens with translucent eggs is disordered, leading to an increased production of lipid peroxides and reducing the antioxidant capacity [34].

### 4.3. Calcium and Phosphorus Content Decreased in Plasma of Hens Laying Translucent Eggs

The primary sources of calcium ions in the blood are from the absorption of nutrients from the intestines. The absorbed calcium is then transported to the uterus, where it is combined with calcium ions to form calcium carbonate, which is deposited onto the eggshell membrane to create the eggshell [35]. Additionally, research has shown that calcium and phosphorus are crucial for eggshell formation, and the levels of these nutrients in the diet are associated with egg translucency [30,36,37]. In our study, the plasma calcium and phosphorus contents of the translucent group were significantly reduced, which also indicated that the laying hens’ plasma calcium and phosphorus contents were associated with the formation of translucent eggs.

### 4.4. Intestinal Digestion, Absorption, and Metabolism Affected Hens Laying Translucent Eggs

The structure of the avian small intestine is fundamental to its digestive and absorptive functions. Metrics such as intestinal villus length and crypt depth provide a comprehensive assessment of intestinal function. Shorter villus length and greater crypt depth can indicate a reduction in the number of mature epithelial cells and a decreased surface area for chyme contact, which may impair nutrient absorption [38,39]. A decreased villus to crypt ratio in the duodenum indicated a damaged mucosa and reduced digestibility in the group of hens laying translucent eggs. Additionally, the activity of intestinal digestive enzymes, such as amylase, chymotrypsin, and lipase, is crucial for nutrient digestion and absorption and is an important indicator of feed utilization efficiency in farm animals [13,40]. We observed that the activity of these intestinal digestive enzymes was lower in the hens laying translucent eggs, particularly chymotrypsin in the duodenum, lipase in the jejunum, and amylase in the ileum. This indicates a diminished intestinal digestibility and absorptive capacity in these hens.

Intestinal AKP plays a pivotal role in maintaining intestinal homeostasis [41]. Our findings revealed a significant decrease in AKP activity in the duodenum of hens laying translucent eggs, which may lead to impaired fat absorption and energy intake. Additionally, Na^+^-K^+^-ATPase and Ca^2+^-Mg^2+^-ATPase are the primary ion pumps responsible for maintaining intracellular and intercellular ion concentration, osmotic balance, transmembrane electrochemical potential, and cellular energy metabolism [42]. We observed a significant reduction in Na^+^-K^+^-ATPase activity in the jejunum and Ca^2+^-Mg^2+^-ATPase activity in the ileum. This indicates that the energy production and utilization in the intestinal tract of laying hens with eggshell translucence are impaired, affecting processes such as the active transport of feed nutrients. Consequently, the formation of translucent eggs may be linked to reduced duodenal mucosal integrity, as well as a diminished nutrient capacity digestion, absorption, and energy metabolism.

### 4.5. Intestinal Antioxidant Capacity Decrease Influenced Hens Laying Translucent Eggs

The balance between the production and utilization of reactive oxygen species (ROS) can be disrupted under pathological or stress conditions, leading to ROS accumulation. T-AOC reflects the comprehensive antioxidant capacity under the co-regulation of CAT, SOD, and GSH-Px. MDA, a toxic lipid peroxidation product formed by unsaturated fatty acids and ROS, indirectly reflect the organism damage [43]. We found a significant decrease in GSH-Px activity in the duodenum, but a significant increase in MDA levels in the same tissue of the group with translucent eggs. This indicates a reduction in intestinal antioxidant capacity, potentially leading to oxidative stress in the intestine, which may impair intestinal Ca^2+^ absorption [44] and affect hens laying translucent eggs.

### 4.6. Transcriptome Analysis in Duodenum

Transcriptome analysis has become one of the most frequently utilized methods in recent research, thanks to the rapid and cost-effective capabilities of Next-Generation Sequencing (NGS) technology, which offers high-throughput gene expression profiling [45]. Our results revealed that the up-regulated genes were predominantly associated with metabolism-related pathways, such as the oxidative phosphorylation pathway. This process mainly occurs in intracellular mitochondria. Mitochondrial dysfunction in the duodenum can impair nutrient and Ca^2+^ absorption, which may result in hens laying translucent eggs.

Glutathione-S transferases (GSTs) play a crucial role in modulating glutathione metabolism. *MGST1*, *GSTA3*, and *GSTT1* are distinct types of GSTs with specific functions. Specifically, *MGST1* has anti-inflammatory and antioxidant effects [46]. *GSTA3*, found in the mitochondria, helps clear various peroxidation products [47,48]. The up-regulation of *GSTA3*, *GSTT1*, and *GSTO2* suggests an enhanced effort to clear peroxide products and alleviating oxidative stress. This indicates that altered intestinal metabolic capabilities could lead to the production of translucent eggs.

## 5. Conclusions

Our study revealed that translucent eggs had a thicker eggshell and lower egg yolk color, which may be attributed to the disruptions in plasma lipid metabolism, decreased intestinal antioxidant capacity, and reduced digestion, absorption, and metabolism capabilities of the laying hens. Our study is also the first to use RNA-seq to identify and annotate DEGs in the duodenal tissues of hens laying translucent versus normal eggs. We identified 327 DEGs associated with translucent egg production, with significant enrichment in pathways related to metabolism, oxidative phosphorylation, and energy homeostasis, including the genes *GSTA3*, *GSTT1*, and *GSTO2*. However, further research is required to fully elucidate the intestinal homeostasis function and the interactions between the microbiota, intestinal epithelium, and host immune system in translucent egg production.

## Figures and Tables

**Table 1:** Egg quality parameters and the chicken plasma biochemical indexes.

Index	Items	Group C	Group T	*p*-Value
Egg quality	Eggshell thickness/mm	0.40 ± 0.04	0.43 ± 0.04	<0.01
	Eggshell ratio/%	10.49 ± 0.92	11.03 ± 0.86	<0.01
	Egg yolk color	9.25 ± 1.96	8.61 ± 1.94	0.01
Plasma biochemical indexes	MDA (nmol/mL)	3.87 ± 0.12	4.56 ± 0.12	0.01
	LDL-C (mmol/L)	5.19 ± 0.60	2.59 ± 0.38	<0.01
	HDL-C (mmol/L)	0.97 ± 0.29	0.37 ± 0.09	0.04
	Calcium (mmol/L)	5.49 ± 1.32	3.16 ± 0.85	0.02
	Phosphorus (mmol/L)	1.60 ± 0.27	1.19 ± 0.30	0.02

Note: Values are expressed as means ± SE. MDA: malondialdehyde; LDL-C: low-density lipoprotein cholesterol; HDL-C: high-density lipoprotein cholesterol.

**Table 2:** Small intestinal morphology and structure, enzyme activity, and antioxidant capacity index.

Tissue	Intestinal	Items	Group C (*n =* 10)	Group T (*n =* 10)	*p*-Value
Duodenum	Morphometry	Villus length/Crypt depth	5.02 ± 0.97	2.61 ± 0.24	0.04
	Digestive enzymes	Chymotrypsin (U/mgprot)	8.75 ± 1.32	7.35 ± 0.42	0.01
	Energy metabolism enzymes	T-ATPase (U/mgprot)	11.55 ± 2.05	9.74 ± 0.92	0.04
		AKP (U/mgprot)	6.19 ± 1.16	5.12 ± 0.72	0.04
		SDH (U/mgprot)	9.89 ± 1.84	7.54 ± 1.07	0.01
	Antioxidant capacity	GSH-Px (umol/gprot)	57.98 ± 4.86	51.40 ± 6.73	0.04
		MDA (nmol/mgprot)	2.09 ± 0.20	2.31 ± 0.10	0.02
Jejunum	Digestive enzymes	Lipase (U/gprot)	6.74 ± 1.02	5.42 ± 0.49	<0.01
	Energy metabolism enzymes	Na^+^K^+^-ATPase (U/mgprot)	1.17 ± 0.11	0.99 ± 0.13	0.01
	Antioxidant capacity	T-AOC (mmol/gprot)	25.31 ± 2.38	21.29 ± 2.73	0.01
		GSH-Px (umol/gprot)	58.52 ± 6.49	52.85 ± 3.91	0.04
Ileum	Digestive enzymes	amylase (U/gprot)	520.94 ± 55.07	449.75 ± 64.96	0.03
	Energy metabolism enzymes	Ca^2+^Mg^2+^-ATPase (U/mgprot)	1.13 ± 0.23	0.90 ± 0.11	0.02

Note: Values are expressed as means ± SE. MDA: malondialdehyde; T-ATPase: total antioxidant capacity; T-AOC: total antioxidant capacity; GSH-Px: glutathione peroxidase; AKP: alkaline phosphatase; SDH: succinate dehydrogenase; Na^+^K^+^-ATPase: natrium potassium ATPase; Ca^2+^Mg^2+^-ATPase: calcium and magnesium ATPase.

## Data Availability

Raw sequence data were deposited in the Genome Sequence Archive in the BIG Data Center, Beijing Institute of Genomics, Chinese Academy of Sciences, and are publicly accessible at http://bigd.big.ac.cn/gsa, accessed on 10 January 2024 (accession no. CRA009523).

## Supplementary Materials

The following supporting information can be downloaded at: https://www.mdpi.com/article/10.3390/ani14172593/s1. Table S1. The gene primer sequences for quantitative real-time reverse-transcription PCR. Table S2. Egg quality parameters, eggshell calcium, phosphorus, ash content, and chicken plasma biochemical indexes. Table S3. Small intestinal morphology and structure, enzyme activity, and antioxidant capacity index. Table S4. Overview of sequencing data quality control. Table S5. Overview of sequencing data mapping rate to the reference genome. Table S6. Statistical interval distribution of FPKM values in samples.
