# Supplementary material for: Plasma Biochemistry, Intestinal Health, and Transcriptome Analysis Reveal Why Laying Hens Produce Translucent Eggs
Source: Animals (Basel). 2024 Sep 6;14(17):2593. doi: 10.3390/ani14172593 (PMC11394436; doi:10.3390/ani14172593)
Supplement: Supplementary file 1 [file animals-14-02593-s001.zip › animals-3141170-supplementary.pdf]

Table S1. The gene primers sequences for quantitative real-time reverse-transcription PCR.

Gene	Accession Number	Primers Sequences（5’-3’）	Primer Length
β-Actin	ENSGALG00000009621	F: GAACCCCAAAGCCAACAG<br>R: GGGCGTAGCCTTCATAGA	182 bp
MGST1	ENSGALG00000013098	F: GGCATTTGCCAACCCAGAAG<br>R: CCTAAAATGCAGCAAGGCCG	199 bp
MGST2	ENSGALG00000009803	F:CGTGTCTCTGCTTTCTGTCCT<br>R:GATTGCAAAACCATCCTGCGA	197 bp
NQO1	ENSGALG00000023437	F:CGCCGAGCAGAAGAAGATTG<br>R:GGTGGTGAGTGACAGCATGG	190 bp
ABCG5	ENSGALG00000009955	F:CCTCGCTCAGAACTCTTCAGG<br>R:CGAGTTCACGTTCCTTGCTTC	196 bp
ABCG8	ENSGALG00000009958	F:GGTAAAAGAGCCCAGGACAGTA<br>R:CCTGAGTTCCAGGGCAATAG	182 bp
F2R	ENSGALG00000014983	F:GACCCGTTTTGTTCCTTCGG<br>R:CCGAGGTCCAAATACCCAGTT	217 bp
FA2H	ENSGALG00000002775	F:AAGTACGACGAGTGGGTGC<br>R:AGTATTTGTGCACGGGGATG	214 bp

Table S2. Egg quality parameters, eggshell calcium, phosphorus, ash content and the chicken plasma biochemical indexes.

Index	Items	Control C	Control T	*P*-value
Egg quality	Egg weight/g	44.97±3.73	45.71±3.56	0.12
	Eggshell index	1.42±1.10	1.31±0.05	0.31
	Eggshell color	9.25±1.96	8.61±1.94	0.51
	Egg yolk ratio/%	34.42±2.27	34.42±2.47	0.98
	Albumen height/mm	4.13±0.71	4.10±0.92	0.80
	Haugh unit	67.71±6.09	66.85±7.65	0.34
	Eggshell strength/(N/cm^2)	43.61±9.06	43.28±10.35	0.80
Calcium, Phosphorus and ash content in eggshell	Calcium/%	33.78±1.23	34.86±0.90	0.50
	Phosphorus/%	0.14±0.007	0.13±0.006	0.40
	Ash content	90.44±2.51	87.82±1.47	0.39
Plasma biochemical indexes	T-AOC/(U/ml)	10.34±2.43	9.98±1.04	0.77
	CAT（U/ml）	9.96±0.65	7.82±0.64	0.07
	GSH-PX	884.23±27.36	836.39±38.58	0.39
	SOD（U/ml）	56.49±1.24	53.79±2.30	0.37
	TG（mmol/L）	26.57±2.19	28.50±1.44	0.50

Note: T-AOC: total antioxidant capacity; CAT: catalase; GSH-PX: glutathione peroxidase; SOD: superoxide dismutase; TG: triglyceride; Ca: Calcium

Table S3. Small intestinal morphology and structure, enzyme activity and antioxidant capacity index.

Tissue	Intestinal	Items	Group C (n = 10)	Group T (n = 10)	*P*-value
Duodenum	Morphometry	Villus length	951.67±79.36	765.65±39.87	0.07
		Crypt depth	202.90±18.80	301.45±26.41	0.16
	Digestive enzymes	Diastase (U/gprot)	545.91±107.18	533.09±82.34	0.79
		Lipase (U/gprot)	5.84±1.31	5.79±1.15	0.93
	Energy metabolism index	$Na^{+}K^{+}$-ATPase (U/mgprot)	1.61±0.72	1.26±0.37	0.22
		$Ca^{2+}Mg^{2+}$-ATPase (U/mgprot)	1.20±0.22	1.11±0.15	0.37
	Antioxidant capacity index	T-AOC (mmol/gprot)	21.38±3.29	20.22±2.27	0.47
		CAT (U/mgprot)	10.59±0.63	10.31±2.70	0.82
		SOD (U/mgprot)	65.62±6.55	62.46±7.41	0.38
Jejunum	Morphometry	Villus length	1051.83±89.04	1058.08±62.40	0.96
		Crypt depth	175.12±7.03	229.96±30.42	0.12
		Villus length/Crypt depth	6.00±0.45	4.97±0.80	0.29
	Digestive enzymes	Diastase (U/gprot)	546.05±84.69	543.41±96.11	0.95
		Chymotrypsin (U/mgprot)	7.28±1.18	7.23±1.14	0.94
	Energy metabolism index	$Ca^{2+}Mg^{2+}$-ATPase (U/mgprot)	1.12±0.09	1.05±0.17	0.26
		T-ATPase (U/mgprot)	10.23±1.04	10.57±0.94	0.54
		AKP (U/mgprot)	5.47±0.72	5.55±0.69	0.84
		SDH (U/mgprot)	7.84±2.12	7.44±1.36	0.66
	Antioxidant capacity index	CAT (U/mgprot)	9.96±1.88	8.78±1.20	0.19
		SOD	56.17±4.28	56.40±3.78	0.91

		(U/mgprot)			
		MDA (nmol/mgprot)	2.31±0.17	2.30±0.35	0.93
Ileum	Morphometry	Villus length	820.72±105.64	706.51±68.50	0.42
		Crypt depth	158.29±12.85	207.19±36.47	0.21
		Villus length/Crypt depth	5.27±0.78	3.63±0.54	0.15
	Digestive enzymes	Chymotrypsin (U/mgprot)	7.08±1.23	7.05±1.00	0.94
		Lipase (U/gprot)	5.54±1.04	5.43±1.02	0.83
	Energy metabolism index	$Na^{+}K^{+}$-ATPase (U/mgprot)	1.02±0.05	1.04±0.24	0.51
		T-ATPase (U/mgprot)	10.23±1.90	9.85±1.22	0.62
		AKP (U/mgprot)	5.43±0.68	5.23±0.80	0.59
		SDH (U/mgprot)	7.18±0.70	6.86±0.98	0.46
	Antioxidant capacity index	T-AOC (mmol/gprot)	21.13±2.78	19.69±2.34	0.32
		CAT (U/mgprot)	10.28±1.99	10.09±3.09	0.88
		GSH-Px (umol/gprot)	52.23±6.07	52.16±2.95	0.97
		SOD (U/mgprot)	54.19±6.11	54.27±4.73	0.98
		MDA (nmol/mgprot)	1.93±0.48	2.08±0.26	0.43

Note: AKP: alkaline phosphatase; $Ca^{2+}Mg^{2+}$-ATPase: calcium and magnesium ATPase; CAT, catalase; GSH-Px: glutathione peroxidase; MDA: malondialdehyde; $Na^{+}K^{+}$-ATPase: natrium potassium ATPase; SDH: succinate dehydrogenase; SOD, superoxide dismutase; T-ATPase: total antioxidant capacity; T-AOC: total antioxidant capacity.

Table S4. Overview of sequencing data quality control

Sample	Raw reads	Base	Clean reads	Base	Ratio %	Q20 %	Q30 %	GC %
N_1	49847702	7.48 G	46829816	7.02 G	93.95	99.88	97.55	52.00
N_2	49817198	7.47 G	45640464	6.85 G	91.62	99.88	97.69	50.50
N_3	54963066	8.24 G	52062936	7.81 G	94.72	99.84	96.75	50.00
N_4	53877008	8.08 G	48664492	7.30 G	90.33	99.88	97.69	51.00
N_5	55070252	8.26 G	47844996	7.18 G	86.88	99.85	97.54	51.00
N_6	50712066	7.61 G	41549494	6.23 G	81.93	99.86	97.58	51.00
N_7	52804110	7.92 G	46274100	6.94 G	87.63	99.88	97.70	51.00
T_1	45971970	6.90 G	42863576	6.43 G	93.24	99.87	97.47	51.50
T_2	55668976	8.35 G	52293940	7.84 G	93.94	99.87	97.73	51.00
T_3	41669192	6.25 G	39217052	5.88 G	94.12	99.89	97.66	51.00
T_4	44604480	6.69 G	41117192	6.17 G	92.18	99.87	97.71	51.00
T_5	42107860	6.32 G	39271816	5.89 G	93.26	99.86	97.54	51.50
T_6	40786044	6.12 G	38508396	5.78 G	94.42	99.88	97.63	51.00
T_7	53127114	7.97 G	50224626	7.53 G	94.54	99.85	97.65	51.50
T_8	53419078	8.01 G	45604422	6.84 G	85.37	99.85	97.45	52.00
T_9	52967082	7.95 G	47086888	7.06 G	88.90	99.85	97.57	51.00

Table S5. Overview of sequencing data mapping rate to the reference genome

**Sample**	VR	MR	UMR	MMR	PEMR	RMS	RMA	NSR	SR
**N_1**	46829816(100%)	37684595(80.47%)	28855369(61.62%)	8829226(18.85%)	34567214(73.81%)	18341508(39.17%)	18597193(39.71%)	26970470(57.59%)	9968231(21.29%)
**N_2**	45640464(100%)	41162836(90.19%)	30921063(67.75%)	10241773(22.44%)	37975384(83.21%)	20039273(43.91%)	20118271(44.08%)	31118346(68.18%)	9039198(19.81%)
**N_3**	52062936(100%)	44869210(86.18%)	34050093(65.40%)	10819117(20.78%)	41328168(79.38%)	21797052(41.87%)	21897401(42.06%)	34045594(65.39%)	9648859(18.53%)
**N_4**	48664492(100%)	43137008(88.64%)	32541593(66.87%)	10595415(21.77%)	39790856(81.77%)	21011916(43.18%)	21129303(43.42%)	32702674(67.20%)	9438545(19.40%)
**N_5**	47844996(100%)	40919172(85.52%)	31551378(65.94%)	9367794(19.58%)	37523254(78.43%)	19885238(41.56%)	20030911(41.87%)	30028077(62.76%)	9888072(20.67%)
**N_6**	41549494(100%)	37400906(90.02%)	28784165(69.28%)	8616741(20.74%)	34404280(82.80%)	18290135(44.02%)	18373201(44.22%)	28934141(69.64%)	7729195(18.60%)
**N_7**	46274100(100%)	40431635(87.37%)	30556391(66.03%)	9875244(21.34%)	37338652(80.69%)	19717636(42.61%)	19834265(42.86%)	29715607(64.22%)	9836294(21.26%)
**T_1**	42863576(100%)	35448064(82.70%)	27207021(63.47%)	8241043(19.23%)	32629922(76.13%)	17272881(40.30%)	17352191(40.48%)	25668030(59.88%)	8957042(20.90%)
**T_2**	52293940(100%)	45427057(86.87%)	34184434(65.37%)	11242623(21.50%)	41974444(80.27%)	22155841(42.37%)	22239519(42.53%)	33646889(64.34%)	10748471(20.55%)
**T_3**	39217052(100%)	34466843(87.89%)	26094925(66.54%)	8371918(21.35%)	32071250(81.78%)	16923480(43.15%)	16951146(43.22%)	25463257(64.93%)	8411369(21.45%)
**T_4**	41117192(100%)	35330798(85.93%)	26717367(64.98%)	8613431(20.95%)	32426970(78.86%)	17212291(41.86%)	17308546(42.10%)	27048775(65.78%)	7472062(18.17%)
**T_5**	39271816(100%)	33343076(84.90%)	24854473(63.29%)	8488603(21.61%)	30887646(78.65%)	16257624(41.40%)	16337901(41.60%)	24331899(61.96%)	8263626(21.04%)
**T_6**	38508396(100%)	32268609(83.80%)	24715368(64.18%)	7553241(19.61%)	29812084(77.42%)	15775380(40.97%)	15831327(41.11%)	22104273(57.40%)	9502434(24.68%)
**T_7**	50224626(100%)	43165945(85.95%)	31557937(62.83%)	11608008(23.11%)	39900622(79.44%)	20867050(41.55%)	21024382(41.86%)	31510881(62.74%)	10380551(20.67%)
**T_8**	45604422(100%)	38646459(84.74%)	29536570(64.77%)	9109889(19.98%)	35513042(77.87%)	18860600(41.36%)	18932458(41.51%)	28406751(62.29%)	9386307(20.58%)
**T_9**	47086888(100%)	41680721(88.52%)	31239775(66.34%)	10440946(22.17%)	38068820(80.85%)	20148115(42.79%)	20297174(43.11%)	31348685(66.58%)	9096604(19.32%)

Note: VR: Valid reads; MR: Mapped reads; UMR: Unique Mapped reads; MMR: Multi Mapped reads; PEMR: PE Mapped reads; RMSS: Reads map to sense strand; RMA: Reads map to antisense strand; NSR: Non-splice reads; SR: Splice reads

Table S6 Statistical interval distribution of FPKM value in samples.

Sample	0-0.1 FI	0.1-0.3 FI	0.3-3.57 FI	3.57-15 FI	15-60 FI	>60 FI
N_1	8083(33.81%)	1309(5.47%)	5346(22.36%)	5723(23.94%)	2757(11.53%)	692(2.89%)
N_2	8547(35.75%)	1337(5.59%)	5580(23.34%)	5179(21.66%)	2587(10.82%)	680(2.84%)
N_3	8170(34.17%)	1304(5.45%)	5139(21.49%)	5570(23.30%)	3041(12.72%)	686(2.87%)
N_4	8356(34.95%)	1317(5.51%)	6034(25.24%)	5242(21.92%)	2309(9.66%)	652(2.73%)
N_5	8570(35.84%)	1306(5.46%)	5279(22.08%)	5299(22.16%)	2710(11.33%)	746(3.12%)
N_6	8617(36.04%)	1331(5.57%)	5746(24.03%)	5107(21.36%)	2453(10.26%)	656(2.74%)
N_7	8505(35.57%)	1271(5.32%)	5324(22.27%)	5334(22.31%)	2742(11.47%)	734(3.07%)
T_1	8773(36.69%)	1361(5.69%)	5348(22.37%)	4933(20.63%)	2659(11.12%)	836(3.50%)
T_2	8255(34.53%)	1354(5.66%)	5487(22.95%)	5310(22.21%)	2696(11.28%)	808(3.38%)
T_3	8345(34.90%)	1249(5.22%)	5068(21.20%)	5520(23.09%)	2940(12.30%)	788(3.30%)
T_4	8537(35.70%)	1374(5.75%)	5689(23.79%)	5257(21.99%)	2432(10.17%)	621(2.60%)
T_5	8348(34.91%)	1259(5.27%)	5105(21.35%)	5374(22.48%)	3021(12.63%)	803(3.36%)
T_6	8563(35.81%)	1253(5.24%)	4902(20.50%)	5087(21.28%)	3074(12.86%)	1031(4.31%)
T_7	8350(34.92%)	1315(5.50%)	5058(21.15%)	5396(22.57%)	3032(12.68%)	759(3.17%)
T_8	8601(35.97%)	1262(5.28%)	5200(21.75%)	5191(21.71%)	2854(11.94%)	802(3.35%)
T_9	8612(36.02%)	1370(5.73%)	5460(22.84%)	5204(21.76%)	2591(10.84%)	673(2.81%)
